# A Single-Culture Bioprocess of *Methanothermobacter thermautotrophicus* to Upgrade Digester Biogas by CO_**2**_-to-CH_**4**_ Conversion with H_**2**_

**DOI:** 10.1155/2013/157529

**Published:** 2013-10-01

**Authors:** Matthew R. Martin, Jeffrey J. Fornero, Rebecca Stark, Laurens Mets, Largus T. Angenent

**Affiliations:** ^1^Electrochaea, LLC, 1005 N. Warson Road, Suite 201, St. Louis, MO 63132, USA; ^2^The University of Chicago, 920 E. 58th Street, Chicago, IL 60637, USA; ^3^Cornell University, 214 Riley-Robb Hall, Ithaca, NY 14853, USA

## Abstract

We optimized and tested a postbioprocessing step with a single-culture archaeon to upgrade biogas (i.e., increase methane content) from anaerobic digesters via conversion of CO_2_ into CH_4_ by feeding H_2_ gas. We optimized a culture of the thermophilic methanogen *Methanothermobacter thermautotrophicus* using: (1) a synthetic H_2_/CO_2_ mixture; (2) the same mixture with pressurization; (3) a synthetic biogas with different CH_4_ contents and H_2_; and (4) an industrial, untreated biogas and H_2_. A laboratory culture with a robust growth (dry weight of 6.4–7.4 g/L; OD_600_ of 13.6–15.4), a volumetric methane production rate of 21 L/L culture-day, and a H_2_ conversion efficiency of 89% was moved to an industrial anaerobic digester facility, where it was restarted and fed untreated biogas with a methane content of ~70% at a rate such that CO_2_ was in excess of the stoichiometric requirements in relation to H_2_. Over an 8-day operating period, the dry weight of the culture initially decreased slightly before stabilizing at an elevated level of ~8 g/L to achieve a volumetric methane production rate of 21 L/L culture-day and a H_2_ conversion efficiency of 62%. While some microbial contamination of the culture was observed via microscopy, it did not affect the methane production rate of the culture.

## 1. Introduction

Organic waste streams contain energy that is stored in biomass, which had originally been harnessed from the sun by photosynthesis. To prevent environmental problems during the release of these waste streams, biological treatment is necessary. At the same time, there is a growing interest in recovering this stored energy in more useful forms by converting the complex biomass into bioenergy sources that are direct replacements of fossil fuels [1]. The traditional route for this conversion with relatively energy-dense wastes is via methane fermentation in anaerobic digesters [2, 3]. Anaerobic digesters consist of an open culture of microbial consortia (referred to here as a reactor microbiome) with a dynamic food web that includes bacterial hydrolysis, acidogenesis, and acetogenesis, as well as archaeal methanogenesis [1].

Anaerobic digestion is an ideal process for two reasons: (1) the product methane bubbles freely out of solution without costly separation; and (2) the anaerobic reactor microbiome harvests the maximum amount of free energy without oxygen by maximizing the production of methane (resulting in high conversion efficiencies) [4]. However, during digestion, both methane and carbon dioxide must be produced to balance the high oxidation number (i.e., number of transferable electrons per carbon) for methane with the low oxidation number for carbon dioxide. The resulting stoichiometric reactions equalize the oxidation state of the products to the oxidation state of the substrate since no alternative electron acceptors or donors are added to an anaerobic system [5]. Therefore, the carbon dioxide content depends on the substrate composition of the organic waste stream, and typically remains within a range of 30–50% [6].

The carbon dioxide in digester biogas is inert as a fuel and dilutes the energy content of the biogas, preventing the introduction of biogas as a renewable natural gas into the current natural gas pipeline infrastructure. With a 30–50% carbon dioxide content, biogas has an energy density of ~18–23 MJ per cubic meter [7], while natural gas has an energy density of 37 MJ per cubic meter. The low energy density of biogas requires modification of energy conversion systems and renders biogas an inefficient energy carrier for long-distance transportation and energy storage [7]. Therefore, most of the biogas is used at or near the point of production to run boilers or combined heat and power systems, such as engines or turbines, or, in a worst-case scenario, it is flared off. Since the electric power efficiencies are low (~35%), a large fraction of the energy is often lost as waste heat when it cannot be directly used in a local setting.

To overcome the low energy content of biogas, three strategies have been developed to upgrade biogas (i.e., increase methane content) into renewable natural gas by: (1) removing carbon dioxide from biogas via postprocessing technologies; (2) supplying a reduced substrate (e.g., hydrogen gas) to the organic waste stream of the anaerobic digester with the goal to convert carbon dioxide into methane *in situ*; or (3) converting carbon dioxide from biogas into methane via postprocessing technologies. The minimum methane content requirement for the product gas to be injected into the natural gas network differs from country to country and depends on specific gas system regulations. For example, within Denmark, the carbon dioxide content of the natural gas cannot exceed 2.5 mol% [8]. For the first strategy, physical gas separation methods (e.g., gas-to-liquid exchange, amine extraction, semipermeable membrane technology, pressure-swing adsorption, or their hybrid variants) have been used at industrial scales to remove carbon dioxide from biogas and to discard it [9–11].

For the second and third strategies, converting carbon dioxide *in situ* or as a postprocessing step, respectively, offers the advantage of simultaneously increasing the net methane production. In a laboratory-scale study, Luo et al. [12] supplied hydrogen gas in addition to a complex organic substrate (manure) to mesophilic anaerobic digesters (second strategy). In essence, they increased the oxidation state of the total substrate (manure and hydrogen gas) to close the gap with the oxidation state of methane, resulting in lower carbon dioxide content in the biogas. The biological hydrogen conversion led to a reduction in carbon dioxide content from 38% to 15% and a methane production increase of 22%. An unanticipated disadvantage was the increase in pH to 8.3, which led to inhibition of methanogenesis [12]. The authors continuously monitored the accumulation of short-chain carboxylic acids, because, thermodynamically, under anaerobic conditions, the oxidation of propionic acid and *n*-butyric acid, which are intermediate chemical species in the anaerobic food web, is not favorable when hydrogen partial pressures reach levels above 10^−2^ kPa [13]. Therefore, regulating the supplementation of hydrogen gas to a constantly varying, complex organic waste stream will be difficult; undersupplementation will lead to an excessive carbon dioxide content in the biogas, while oversupplementation will lead to accumulation of short-chain carboxylic acids and unstable digester conditions with a reduced overall methane yield.

For the third strategy of upgrading biogas in a postprocessing step, an abiotic or biological system could be used. For another possibly carbon-dioxide-rich industrial gas (i.e., synthetic combustion gas), Hoekman et al. [14] had explored the use of metal catalysts to convert carbon dioxide into methane. In addition, several pure-culture methanogens, including *Methanothermobacter thermautotrophicus*, were tested to convert carbon dioxide from a synthetic fermentation off gas into methane [15]. For biogas upgrading, Luo and Angelidaki [16] operated a thermophilic, laboratory-scale anaerobic digester while continuously feeding a synthetic biogas stream that consisted of 60% H_2_, 25% CH_4_, and 15% CO_2_ (4 : 1 ratio of H_2_ : CO_2_) and achieved a maximum methane content in the product gas of ~95%. Over the operating period of one month, the reactor microbiome (open culture) in this digester became enriched with hydrogenotrophic methanogens, but other trophic groups remained active, such as homoacetogenic bacteria. In addition, a microbiome characterization found a diverse group of thermophilic, anaerobic methanogens, including a sequence with a 93% ID to *M. thermautotrophicus* [16]. In another study with CO gas fed into an anaerobic digester, sequences with a <93% ID to *M. thermautotrophicus* were abundant [17]. In all these studies, a synthetic gas was used rather than an industrial biogas.

*M. thermautotrophicus* is a lithoautotrophic, thermophilic (40–70°C) methanogenic archaeon, which was first isolated as strain deltaH from sewage sludge at a wastewater treatment facility in Urbana, IL [18], and has a sequenced genome [19]. *M. thermautotrophicus* deltaH was described as a strict, obligate anaerobe with an optimal growth temperature of 65–70°C and pH of 7.2–7.6 [18]. Other related strains have been isolated, including strain Hveragerdi, which was isolated from an Icelandic alkaline hot spring [20]. *M. thermautotrophicus* conserves energy by using hydrogen to reduce carbon dioxide to methane and also uses carbon dioxide as its carbon source.

Some knowledge with laboratory-scale bioprocessing with a pure culture of *M. thermautotrophicus* strain Hveragerdi has been published in two studies by Schill et al. [21, 22]. The authors found that growth of *M. thermautotrophicus* with a continuous H_2_ : CO_2_ (4 : 1) and medium flow was not just dependent on the dilution rate, which is commonly accepted for chemostats with a liquid substrate, but that both gas influent rates and dilution rates needed to be taken into account. In addition, they observed and modeled that the gaseous substrate consumption (removal) rates positively influenced the effective gas transfer flux into the system by maintaining a low effective gas concentration. A higher gas transfer flux resulted in a considerably higher production rate than when modeled with a standard gas transfer coefficient (*k*_*L*_ *a*) and the bulk liquid concentration (*c*_*D*_) values for the gaseous substrate with the lowest solubility. The highest *k*_*L*_ *a* reported for this study was 2,300 h^−1^ with a biomass concentration of 4.84 gL^−1^ at their highest gas flow rates. The authors also found a hydrogen conversion efficiency (into methane and biomass) of 88% at their lowest gas flow rates [21]. In addition, thermodynamic calculations explained why heat dissipation will be higher than for other bioprocesses: (1) cell synthesis from carbon dioxide will require a high energy expense; and (2) the entropy will drop considerably because the small molecules of hydrogen and carbon dioxide are converted to macromolecules [23]. The same authors further refined their mathematical model and experiments and observed that the growth rates for *M. thermautotrophicus* have to be very small with a large heat production rate, which they called entropy-retarded growth [22].

Biogas upgrading strategies that utilize living microbes to convert carbon dioxide into methane have the advantage of relying on a self-replicating catalyst. To prevent conversion of hydrogen gas into acetate by homoacetogens, and a resulting efficiency loss of biogas upgrading, the inoculation of the bioprocess with a pure-culture archaeon will be advantageous compared to a reactor microbiome. Next, to be of practical use in industrial settings, the ideal archaeon inoculum must also be able to withstand: (1) accidental exposure to oxygen; (2) exposure to hydrogen sulfide (which is often present in biogas); (3) contamination from other bacteria or phages, which are present in the continuously fed biogas; (4) possible inhibition by a high methane content in biogas; and (5) intermittent supply of renewable hydrogen due to the fluctuating nature of wind and photovoltaic energy sources. A robust archaeon must also avoid byproduct production and enable a highly energy efficient process. Here, we studied biogas upgrading with a pure culture of *M. thermautotrophicus *fed with hydrogen gas in combination with biogas. We optimized bioprocessing and tested biogas upgrading by performing four experiments: Experiment 1 with a synthetic H_2_/CO_2_ gas without methane; Experiment 2 with a synthetic H_2_/CO_2_ gas without methane and a pressurized headspace; Experiment 3 with a synthetic biogas; and Experiment 4 with an industrial, untreated biogas from the Anheuser-Busch InBev facility in St. Louis, MO. Our findings suggest that biogas can be upgraded with a pure culture of *M. thermautotrophicus *fed by an external source of H_2_ gas and that the function of a *M. thermautotrophicus *culture was maintained during an 8-day operating period using an untreated industrial biogas even though some microbial contamination was observed.

## 2. Materials and Methods

### 2.1. Archaeal Strain and Growth Conditions

For the experiments described here we obtained an evolved *M. thermautotrophicus *strain from the Mets Lab (University of Chicago, IL), which was originally procured as *M. thermautotrophicus* strain Hveragerdi (DSM 3590) [20] and adapted in the laboratory to long-term (>1 year) stable methane production under slow growth conditions (doubling times >7 days). The liquid 25x medium consisted of: 250 mM KH_2_PO_4_; 250 mM NaCl; 25 mM MgCl_2_-6H_2_O; 20 mM Na_3_ nitrilotriacetate; 10 mM nitrilotriacetic acid; 12.5 mM L-cysteine; 5 mM FeSO_4_-H_2_O; 0.25 mM Na_2_WO_4_; 0.125 mM NiCl_2_-6H_2_O; 0.0625 mM CoCl_2_-6H_2_O; 0.0625 mM Na_2_MoO_4_-2H_2_O; 0.05 mM resazurin; and 0.025 mM Na_2_SeO_3_ (Sigma Aldrich, St. Louis, MO). Medium and other reagents were prepared with water that was boiled with the goal to sterilize and remove oxygen. For initially growing the culture in the bioreactor, a 1x medium was prepared by dilution (4% 25x medium with 96% water). Since our culture generated metabolically produced water, the active volume increased during the operating period. The increased volume was pumped intermittently from the bioreactor and replaced simultaneously by the 1x medium. As a source of sulfur, 0.63 mL/h Na_2_S (Sigma Aldrich) solution at a concentration of 500 mM was provided by a continuous feed to the culture (H_2_S is formed in solution, but it is continuously lost with the effluent gas), except during feeding of industrial biogas, which included H_2_S (~7,000 ppm). The temperature of the active volume was maintained at 60°C. The pH of the culture was controlled at 6.85 via ammonium hydroxide addition, which also provided the nitrogen source for *M. thermautotrophicus*. In Experiment 3, at a methane influent rate of 0.2 L/min, the pH was maintained at 7.35. During all experiments, except for when industrial biogas was used, cultures were continuously purged with a gas mixture that consisted of 4 : 1 H_2_ : CO_2_ (Cee Kay gas, St. Louis, MO). For baseline conditions before Experiments 1, 2, and 3, the total influent gas rate was maintained at 0.5 L/min by feeding 0.4 L/min H_2_ and 0.1 L/min CO_2_. Before Experiment 4, this was 0.25 L/min by feeding 0.2 L/min H_2_ and 0.05 L/min CO_2_. For both the synthetic H_2_ : CO_2_ gas mixture experiments (Experiments 1 and 2), the H_2_ influent rate was varied (0.1, 0.2, 0.4, 0.8, and 1.6 L/min for Experiment 1; and 0.4, 0.48, 0.8, 0.96, 1.6, and 1.99 L/min for Experiment 2), while maintaining the 4 : 1 H_2_ : CO_2_ gas mixture ratio. For the synthetic gas mixture experiment with pressure (Experiment 2), we increased the headspace pressure inside the bioreactor from 101 kPa (atmospheric pressure) to 122 kPa by routing the product gas through a 2.1 m vertical water column. For the synthetic biogas experiment (Experiment 3), we utilized an experimental design with three different methane influent rates (0, 0.2, and 0.4 L/min) for three different hydrogen influent rates (0.2, 0.4, and 1.6), resulting in nine different conditions with different methane contents (0–62%) and total flow rates (0.25–2.4 L/min) in the influent gas (Table 1). Finally, for the industrial, untreated biogas (Experiment 4), we maintained the hydrogen influent rate at 0.2 L/min throughout the operating period. For the first 7 days (164 h) of Experiment 4, we fed 0.05 L/min of CO_2_, which we replaced with industrial biogas for the final 8 days (192 h; from 164 to 356 h of the operating period).

### 2.2. Experimental Setup and Operating Conditions

**Figure 1:**
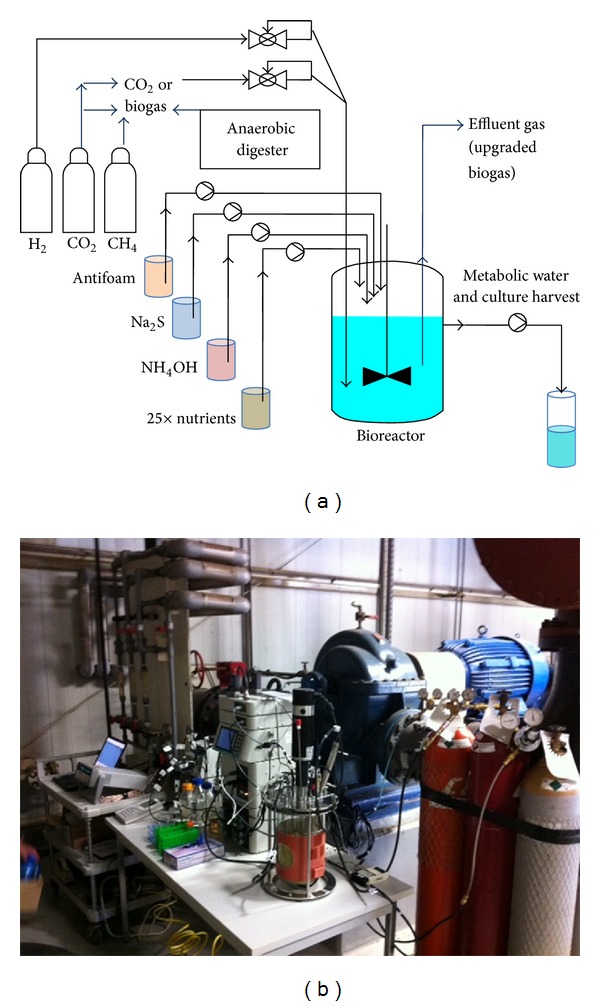
Schematic of the bioreactor setup (a) and view of this setup at the industrial site (b).

The experiments were performed in a bioreactor (BioFlow 110, New Brunswick Scientific, Enfield, CT) with three, 6 cm ID Rushton-type impellers at fluid heights of 1, 2, and 3 L and a downdraft-type impeller (New Brunswick) at the surface of the medium (Figure 1(a)). The bioreactor was stirred continuously at 700 rpm with an active culture volume of ~3.5 L (Experiment 1) and 3.0 L (Experiments 2, 3, and 4). We installed the baffles and the ring sparger that were included with the bioreactor setup (New Brunswick). The rates of supply for synthetic gases from cylinders were both controlled and recorded using digital mass flow controllers (EW-32907-69, Cole-Parmer, Vernon Hills, IL), and total gas effluent rates were measured with a custom-made 0.5 L soap bubble flow meter. Temperature, pH, ORP, and fluid level in the reactor were monitored continuously; the temperature was maintained using an external heating jacket, and the pH was controlled by adding a 2 M solution of NH_4_OH (Sigma-Aldrich). Antifoam (SE-15, Sigma-Aldrich) was fed continuously into the reactor at a rate of 0.30 mL/h  to eliminate foam. For the pressure experiment (Experiment 2), we installed a 5.1 cm diameter PVC pipe for the vertical water column with the product gas line at the bottom. At the industrial digester facility, unfiltered biogas was fed into the bioreactor via 15 m tubing (Norprene L/S 17, Cole-Parmer). For the laboratory-scale experiments, once anaerobic conditions were established inside the bioreactor (as determined by ORP readings and the absence of color of the resazurin indicator), the media was inoculated with 1 mL of a pregrown *M. thermautotrophicus* culture. The 1x medium solution was fed intermittently. The simultaneous feeding and decanting was performed automatically when the liquid level surpassed a preset active volume. The volume of the decanted liquid was measured at regular intervals. For the laboratory-scale experiments, dry weight and OD_600_ were monitored daily to determine the rate of culture growth and to determine if steady-state conditions were achieved.

### 2.3. Operating Conditions at the Industrial Anaerobic Digester Facility

During Experiment 4, after a 5-day (117 h) operating period, the steady-state culture in the laboratory was shut down overnight to cool down before the ~30 min transport to the industrial facility (Figure 1(b)). We chose the anaerobic digester facility at the Anheuser-Busch InBev brewery in St. Louis, MO, where brewery wastewater is treated with six expanded granular sludge bed-Biobed systems. Immediately upon arrival at the industrial facility, the reactor was redeployed. Temperature, pH, and fluid level controls were reactivated, and the reactor was supplied with synthetic gas at the same flow rates that were used for the laboratory operating conditions. After the reactivation of the bioreactor, we monitored culture OD_600_, dry weight, and influent and effluent gas flows more closely for ~24 h to ensure that the culture and conversion efficiencies were stable following the move. Next, during the feeding of industrial and unfiltered biogas instead of the synthetic CO_2_ gas flow and Na_2_S solution feeding (H_2_S in biogas supplied the sulfur source), we maintained all other operating conditions similar to those used in the laboratory (e.g., temperature, pH, H_2_ feeding flow, and automatic and intermittent medium flow). The biogas influent rate was controlled by a peristaltic pump (Cole-Parmer, Vernon Hills, IL) and was set to 0.24 L/min to minimize the risk that CO_2_ would be unintentionally limiting (based on a ~25% carbon dioxide content in the biogas). Influent and effluent gas flow rates were measured by soap bubble measurement throughout the experiment. In addition, culture samples were taken daily for optical density, dry weight analysis, and visual observation via light microscopy. Samples of the influent biogas and effluent gas were collected at hour 166 and 331 of the operating period.

### 2.4. Analytical Procedures

Dry weight of biomass was determined by using a centrifuge (14,000 ×g) to pellet triplicate, 1 mL culture samples, decanting the excess liquid, and drying the pellet at 60°C until a stable weight reading was attained. For OD_600_ analysis, 2-fold serial dilutions were made of the culture, and the absorbance of each was recorded in a 1 cm path length with 600 nm light. Dilutions were chosen such that 3-4 consecutive dilutions gave OD_600_ readings between 0.05 and 0.8. Next, an OD_600_ of the undiluted culture was calculated. Samples were observed at 400x magnification using a light microscope (DMI4000B, Leica, Buffalo Grove, IL) with care taken to observe either the presence of contamination or any morphological changes to the organism, and images were captured using Leica Application Suite Software. Gas samples were collected in evacuated 1 L Summa canisters and analyzed by TestAmerica Laboratories (Costa Mesa, CA). The gas samples were analyzed with a gas chromatography standard test for CO_2_, CO, H_2_, CH_4_, O_2_, and N_2_ (according to ASTM D1946).

### 2.5. Estimating Conversion Efficiencies

The H_2_ conversion efficiencies and CH_4_ production rates of the culture were calculated by (1) and (2), respectively. Assuming the conversion of 5 volumes of influent gas (4 volumes of H_2_ and 1 volume of CO_2_) to 1 volume of effluent gas (CH_4_) with the assumption that the only considerable sink for H_2_ is methanogenesis (4H_2_ + CO_2_→ CH_4_ + 2H_2_O) [21], we expressed the H_2_ conversion efficiency:

(1) $$\begin{array}{c} {\text{H}}_{2}\;\text{conversion}\;\text{efficiency}\;\left(\%\right)=\frac{V_{\text{in}}-V_{\text{out}}}{V_{{\text{H}}_{2}}}*100\%, \end{array}$$

where *V*_in_ = the total flow rate of influent gas; *V*_out_ = the total flow rate of effluent gas; and *V*_H_2__ = the influent flow rate of H_2_ (i.e., H_2_ influent rate). The CH_4_ production rate of the culture was then calculated as (if we assume that the carbon dioxide utilization as a carbon source for growth is small):

(2) CH4  production  rate  =  H2  conversion  efficiency  ×  CO2  influent  rate100%.

## 3. Results

We operated a similar bioreactor setup with a single-culture of *M. thermautotrophicus* for four different experiments: Experiment 1 with a synthetic H_2_/CO_2_ mixture; Experiment 2 with the same synthetic gas mixture but with a pressurized bioreactor headspace; Experiment 3 with H_2_ gas and a synthetic biogas at different CH_4_ contents; and Experiment 4 with H_2_ gas and an untreated biogas from an industrial facility. To test the behavior of the culture to untreated biogas with its microbial and chemical contaminations, we placed the culture at an industrial brewery facility for an operating period of 8 days. During this operating period, the health and density of the culture was monitored, in addition to the methane composition of the product gas stream exiting the *M. thermautotrophicus* bioreactor. The purpose of Experiment 4 was to: (1) evaluate whether the culture of *M. thermautotrophicus* was able to utilize biogas as a CO_2_ source for methanogenesis; and (2) evaluate any response of the *M. thermautotrophicus* culture to biological and chemical contaminants in the biogas stream.

Before starting the experiments that are described here, we knew from preliminary work and from Schill et al. [21], who used a 1.5 L culture with a mixing rate of 1,000 rpm, that the hydrogen mass transfer flux would be limiting in our system and not the catalytic activity of the culture (hydrogen has a considerably lower gas transfer coefficient than carbon dioxide). This resulted in higher volumetric methane production rates (VMPRs) when the mixing speed was increased from 0 to 1,200 RPM in our bioreactor setup (data not shown). To compare results between experiments, we operated the bioreactor with a constant mixing rate of 700 rpm and with a single gas sparger ring. Further improvements to increase the gas transfer flux rates and the VMPRs are, therefore, attainable. However, a careful compromise between optimum performance and economic operating conditions must be made.

### 3.1. Experiment 1: Feeding a Synthetic H_**2**_/CO_**2**_ Mixture

During Experiment 1, we varied the hydrogen influent rate between 0.1–1.6 L/min during each of four days to investigate the effect on methane production rates and hydrogen conversion efficiencies. This experiment was performed with a culture that was grown to steady-state conditions with a dry weight of ~11 g/L at a hydrogen influent rate of 0.4 L/min. With a hydrogen influent rate of 0.4 L/min and an assumed biomass composition for Archaea (CH_1.68_O_0.39_N_0.24_) as discussed by Schill et al. [21], we estimated with a direct measure of the biomass production as part of a mass balance experiment, which we performed over a period of 43 h, that 1.4% of the carbon in carbon dioxide was diverted to growth. During the rest of the study, we assumed this small growth to be negligible during the calculations of the hydrogen conversion efficiencies and methane production rates.

**Figure 2:**
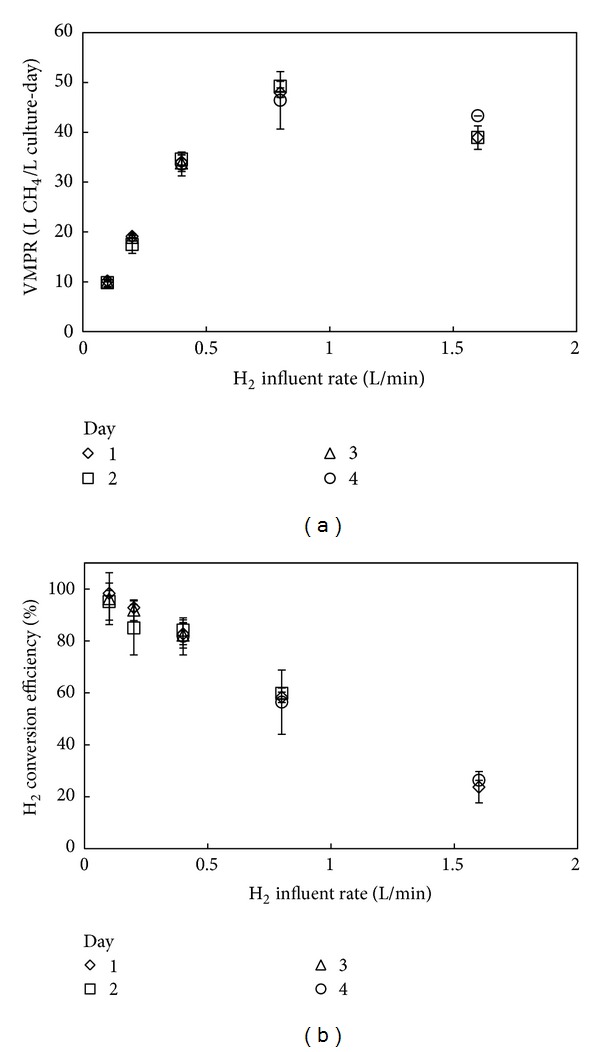
Bioreactor performance for Experiment 1. Volumetric methane production rates (a) and hydrogen conversion efficiencies (b) for bioreactor runs in which the hydrogen influent rate was varied. Data were gathered during four days. The error bars represent the observed variance between runs for each bioreactor condition within one day of experimentation.

We observed that the VMPR achieved an average maximum rate of 47.9 L/L culture-day (SD = 1.40; *n* = 3; Table 2), which was observed at a hydrogen influent rate of 0.8 L/min (Figure 2(a)). A further increase in the hydrogen influent rate did not increase the VMPR. The increase in hydrogen addition from 0.1 to 1.6 L/min to the bioreactor resulted in an almost linear decline in the hydrogen conversion efficiency (Figure 2(b)). As anticipated, the average maximum efficiency of 96.6% (SD = 1.60%; *n* = 3; Table 2) was achieved at the lowest hydrogen influent rate, while this was vice versa at the highest hydrogen influent rate (24.5%; SD = 1.54%; *n* = 3; Table 2). Thus, higher feeding rates of hydrogen gas (and carbon dioxide gas because the 4 : 1 H_2_ : CO_2_ ratio was constant) increased hydrogen fluxes and methane production rates (note that a maximum methane production was observed at the penultimate hydrogen influent rate for atmospheric pressures) at the expense of less efficient H_2_ conversion.

### 3.2. Experiment 2: Pressurizing the Headspace

**Figure 3:**
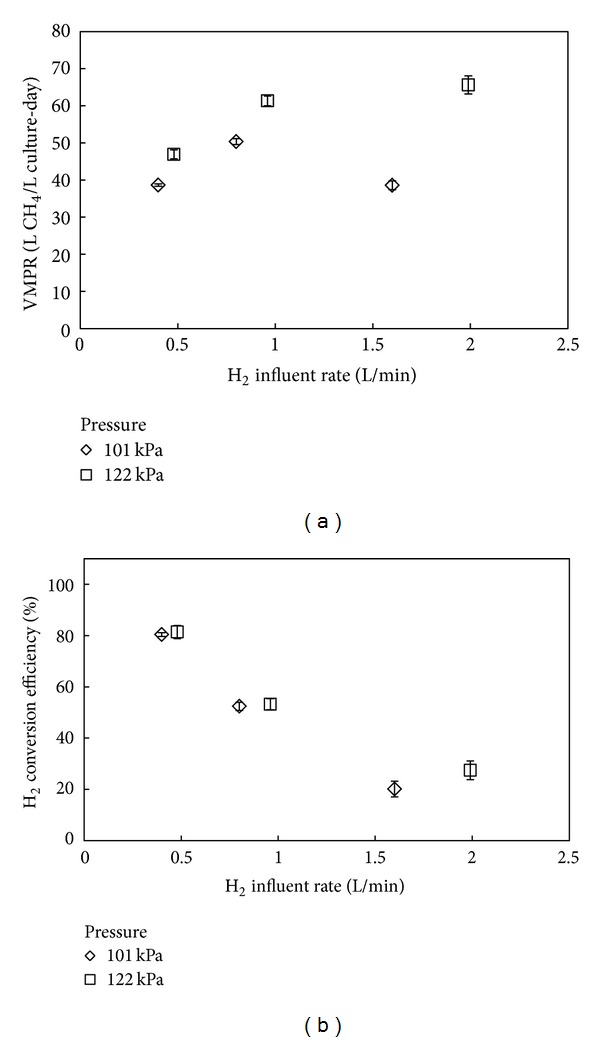
Bioreactor performance for Experiment 2. Volumetric methane production rates (a) and hydrogen conversion efficiencies (b) for bioreactor runs in which the hydrogen influent rate was varied. Data were gathered during two different headspace pressures in the bioreactor (atmospheric pressure (101 kPa) and 122 kPa). The error bars represent the observed variance between runs for each bioreactor condition.

During Experiment 2, we compared the results that were obtained from Experiment 1 with and without pressurization of the bioreactor headspace (101 kPa and 122 kPa, resp.). We anticipated that the bioreactor performance would improve considerably because pressurization increases the hydrogen partial pressures that control the limiting hydrogen mass transfer flux. The experiment was performed with a culture that was pregrown to a dry weight of 11 g/L at a hydrogen influent rate of 0.4 L/min. First, we obtained similar VMPRs between Experiment 1 and the atmospheric pressure condition for Experiment 2 (Figure 3(a)), although it became more apparent that the higher H_2_ influent rate of 1.6 L/min resulted in a lower VMPR compared to an H_2_ influent rate of 0.8 L/min (Figures 2(a) and 3(a)). We explain that the lower VMPRs at the higher H_2_ influent rates is due to hydrogen mass transfer limitations because of reduced hydrogen residence times, which we discuss further in the implementation section that follows. Third, the maximum VMPR was increased to 65.6 L/L-day for the pressurized system (Figure 3(a) and Table 3), which was also the maximum VMPR achieved during this study, albeit it was at the highest applied hydrogen influent rate of 1.99 L/min. Pressurization also improved the low H_2_ conversion efficiencies that we observed for the highest H_2_ influent rates (Figure 3(b)), as anticipated from the increases in VMPRs.

### 3.3. Experiment 3: Feeding a Synthetic Biogas Mixture

**Figure 4:**
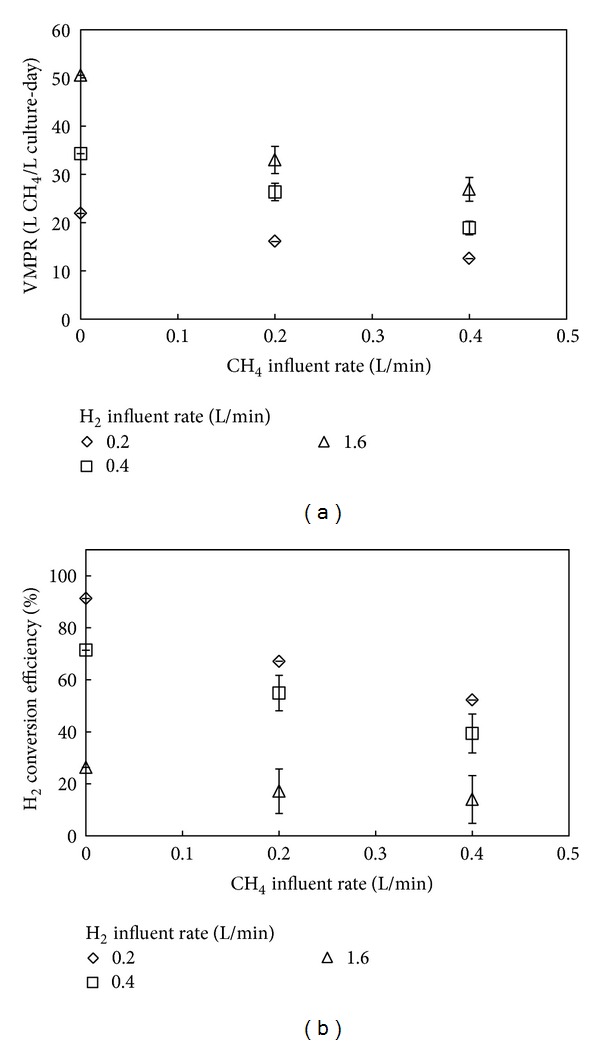
Bioreactor performance for Experiment 3. Volumetric methane production rates (a) and hydrogen conversion efficiencies (b) for bioreactor runs in which the methane influent rate was varied between 0 and 0.4 L/min. Data were gathered for three different hydrogen influent rates. The error bars represent the observed variance between runs for each bioreactor condition.

During Experiment 3, we varied the methane influent rate in the synthetic biogas for three different hydrogen influent rates to investigate the effects of the presence of methane in the influent gas on the bioprocess. We performed this experiment with a culture that was grown to steady-state conditions with a dry weight of 4.1 g/L at a hydrogen influent rate of 0.4 L/min. The maximum VMPR of 50.5 L/L-day for Experiment 3 (Figure 4(a)) was achieved with the highest hydrogen influent rate of 1.6 L/min without adding methane. The addition of methane at 0.4 L/min reduced the VMPR considerably to 26.9 L/L-day, which constituted a 47% decrease in methane production (Figure 4(a)). For the lowest hydrogen influent rate of 0.2 L/min, the VMPR without any methane in the influent gas was lower (21.9 L/L-day) than at a hydrogen influent rate of 1.6 L/min, as anticipated from Experiment 1. The introduction of a methane influent rate of 0.4 L/min resulted in a lower VMPR of 12.6 L/L-day (Figure 4(a)), which constituted a 43% decrease in methane production. This was a slightly lower decrease for the condition with 0.2 L H_2_/min compared to the 1.6 L H_2_/min even though the methane content was much higher (62% versus 17%, respectively; Table 1). For the middle hydrogen flow rate condition of 0.4 L/min (maximum methane content of 44%; Table 1), the difference between the maximum and minimum VMPR for a methane influent rate of 0 and 0.4 L/min was 45%, which fell in between the 47% and 43% decreases of the highest and lowest hydrogen influent rates. This observation shows that a careful consideration of both flow rate and methane content for the influent gas is necessary rather than only the methane content. We observed the relative weakest effect of introduction of methane influent flow to the VMPR for the conditions with the lowest hydrogen influent rate of 0.2 L/min.

For all conditions, though, an increase in methane content led to a lower VMPR (Figure 4(a)), but this was not caused by product inhibition of methane because the largest decrease in VMPR was observed with the lowest methane content of 17% in the influent gas with the highest total influent rate. Therefore, methane in the influent gas reduced the hydrogen partial pressure by dilution, and this led to a decrease in the mass transfer flux of hydrogen that controlled the archaeal conversion rates. Similar to Experiment 1, the hydrogen conversion efficiency showed an opposite behavior compared to the VMPR (Figure 4(b)). Thus, the strongest effect of methane influent flow introduction to the hydrogen conversion efficiency was observed for the lower hydrogen influent rate of 0.2 L/min.

### 3.4. Experiment 4: Feeding Untreated Biogas

**Figure 5:**
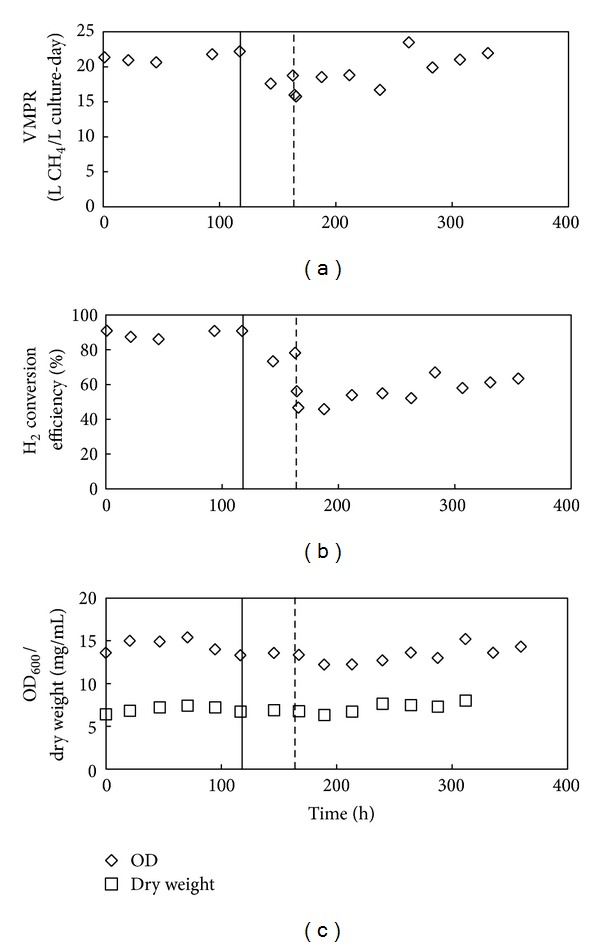
Bioreactor performance for Experiment 4. Volumetric methane production rates (a), hydrogen conversion efficiencies (b), and biomass concentrations (c) during one bioreactor run. The bioreactor was moved from the laboratory to the industrial site on hour 117 of the operating period (straight line) while untreated biogas was introduced instead of synthetic CO_2_ on hour 164 of the operating period (dotted line).

During Experiment 4, after 164 h of the operating period (Figure 5), we switched from a synthetic H_2_/CO_2_ gas mixture to an untreated biogas influent stream with a synthetic H_2_ influent stream to evaluate the effect of industrial conditions on the *M. thermautotrophicus* culture. Experiment 3 had informed us to use the lower hydrogen influent rate of 0.2 L/min because this flow rate would affect the VMPR the least by the presence of methane in the biogas. After obtaining a steady-state culture, we operated the culture for 5 days (117 h) in the laboratory while monitoring its function closely. During this period (0–117 h), the VMPR and H_2_ conversion efficiency remained constant at 21.4 L/L culture-day (SD = 0.6; Figure 5(a)) and 89.1% (SD = 2.3%; Figure 5(b)), respectively, while the dry weight concentration and the OD_600_ fluctuated between 6.4 and 7.4 g/L and 13.6 and 15.4, respectively (Figure 5(c)). The move to the industrial setting, which included not feeding the culture overnight and cooling it down to room temperature, reduced the performance slightly for the next day when operating conditions had been restored, including the temperature of the bioreactor mixed liquor. We observed a recovery on the second day after commencement of the similar conditions as in the laboratory (between 117 and 164 h). Next, after the switch to biogas on hour 164  of the operating period, the dry weight of the culture initially decreased slightly before stabilizing to an increased level of 8.0 g/L (OD_600_ = 15.2; Figure 5(c)) to achieve a volumetric methane production rate of 21.0 L/L culture-day (SD = 1.0; Figure 5(a)) and a H_2_ conversion efficiency of 61.9% (SD = 4.5%; Figure 5(b)) between 283 and 331 h of the experiment. We showed that an insignificant, but slightly lower, VMPR was achieved with industrial biogas compared to synthetic CO_2_ but at a significant lower hydrogen conversion efficiency (as anticipated from Experiment 3 due to dilution with methane).

**Figure 6:**
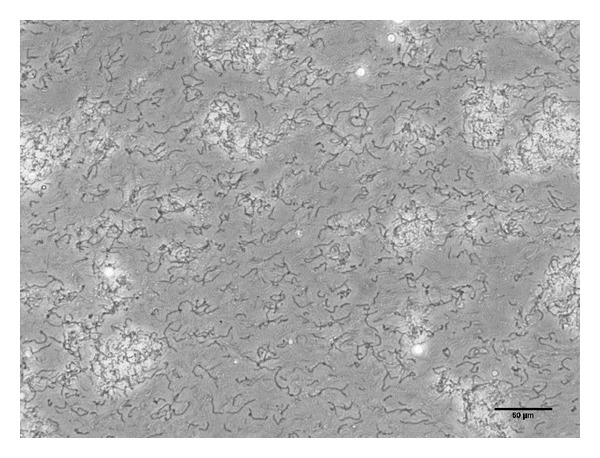
Microscopy view of the *M. thermautotrophicus* culture. The filamentous morphology of the methanogens remained vastly abundant; however, small microbial contaminants were observed. The bar is equal to 50 *μ*m.

Immediately after switching to unfiltered biogas, the culture showed slightly lower VMPRs, but the performance improved during the final 192 h of the operating period, and no long-term inhibition of one or more components in the biogas to *M. thermautotrophicus *was observed based on the VMPR data. Microscope imaging of samples taken from the reactor indicated that a minor biological contamination with a rod-shaped morphology had occurred, but that the vast majority of the cells consisted of the long, thin filaments, which represent the characteristic morphology of *M. thermautotrophicus*, in what appeared to be an almost single-culture population (Figure 6). The microbial contamination, which was not identified, did not affect the efficacy of the culture during the operating period of 8 days.

Gas composition samples were taken shortly after the switch to industrial biogas (hour 166) and at hour 331 of the operating period. For these two time points, the carbon dioxide content in the effluent gas was 16% (versus 30% in the biogas) and 13% (versus 28% in the biogas), respectively. Even though an improvement in the gas quality was achieved in regards to carbon dioxide content, optimizations in how the way the system must be operated would be needed to ensure on-specification renewable natural gas. The methane content for these two time points was actually lower (54% versus 68% in biogas and 61% versus 66% in biogas,resp.) due to dilutions with hydrogen gas (the hydrogen content was 33% and 28%, resp.) because of low hydrogen conversion efficiencies of 46.6% and 61.0%, respectively (Figure 5(b)). We modeled the calculated H_2_, CO_2_, and CH_4_ contents in the effluent gas from the hydrogen conversion efficiency data that were estimated with (1). The best fit (lowest Chi square) between the estimated and measured H_2_, CO_2_, and CH_4_ contents in the effluent gas was found with hydrogen conversion efficiencies of 46.7% and 59.4%, respectively, which are in good agreement with the estimated efficiencies of 46.6% and 61.0%, respectively.

## 4. Discussion

### 4.1. Comparisons between Experiments and Other Studies

Pure-culture growth in continuous bioreactors with gaseous substrates is a function of the liquid medium dilution rate as well as the gas influent rate [21]. With relatively low dilution rates, this resulted in a very high biomass concentration for *M. thermautotrophicus* of ~5 g/L in Schill et al. [21] and in our Experiment 4 (with a hydrogen influent rate of 0.2 L/min and with industrial, untreated biogas) for which we achieved a dry weight concentration and OD_600_ of 8.0 g/L and 15.2, respectively. Both single-culture studies (Schill et al. [21] and our study) showed biomass concentrations that were considerably higher than what is typically observed in chemostats with a liquid influent. As a result, the high hydrogen uptake rates of the robust biocatalyst resulted in a relatively high hydrogen gas transfer coefficient with hydrogen as the limiting gas, resulting in the superior effective gas transfer flux that supported the observed maximum VMPR of 163 L/L culture-day (4.39 g/L-h) (Table 3) [21]. Our maximum VMPR with a H_2_/CO_2_ gas mixture was ~1/3 of this rate (Table 3). According to Experiments 1 and 2, the hydrogen gas transfer flux was limiting the conversion rate of our bioprocess. Therefore, the differences in operating conditions between the studies, such as a larger active volume of 3 versus 1.5 L and a lower mixing speed of 700 versus 1,000 rpm, resulted in considerably lower power to volume rations, causing less mixing activity, which explain the lower hydrogen gas fluxes that we observed compared to Schill et al. [21, 22]. Because of these large effects on the hydrogen transfer flux by changing the operating conditions, care must be taken to maintain high conversion rates during scale up while limiting the parasitic energy input of mixing.

Within our study, we found little difference in VMPRs of the *M. thermautotrophicus* culture whether it was fed with industrial biogas or synthetic biogas (Table 3). Thus, upgrading of biogas shows promise for application in industrial biogas production sites. We did, however, observe a considerable drop in VMPR from ~50 to ~12 L/L culture-day when the influent gas stream contained methane (Table 3). The experimental results are consistent with a role for methane as an inert gas in the system by increasing the gas flow through the reactor, decreasing the gas residence time, and decreasing the hydrogen gas transfer flux into the medium. This influence of methane as a diluent of the reactive gases (H_2_ and CO_2_) must be considered in the engineering design of industrial postprocessing biogas upgrading bioreactors.

The VMPR of 12 L/L-day (0.3 g/L-h) is similar to rates that have been achieved with high-rate anaerobic digesters for easily degradable organic wastewater (Table 3) with very high volatile suspended solids concentrations of 50 g/L due to biofilm (granular biomass) formation, even though the methanogens existed in a very diverse reactor microbiome [24]. Because of the need for high mixing intensities to overcome hydrogen transfer flux limitations, such high biomass concentrations via biofilm formation are not anticipated for our bioreactor system with gaseous substrates. Feeding hydrogen gas into a low-rate anaerobic digester with a reactor microbiome to upgrade biogas did not result in compatible VMPRs when compared to the single-culture studies with *M. thermautotrophicus* (Table 3). A lower, but compatible, VMPR of 5.3 L/L/-day (0.14 g/L-h) was achieved by Luo and Angelidaki [16] with a postprocessing bioreactor system to upgrade biogas with an acclimated reactor microbiome of enriched thermophilic hydrogenotrophic methanogens (Table 3). It is not clear at this point whether a further acclimated microbiome will eventually be able to achieve maximum VMPRs as high as observed with our single-culture of *M. thermautotrophicus* fed with biogas. Although, we anticipate that the observed acclimated homoacetogens in the reactor microbiome by others [16] would reduce the efficiency considerably, failing to ever achieve the performance of the pure culture archaeon. It is noteworthy that the study by Luo and Angelidaki [16] used a smaller active volume of 600 mL and a higher mixing intensity of 800 rpm than our study. The power to volume ratios were, therefore, closer to the study by Schill et al. [21] than to our study, while Schill et al. [21] obtained a far superior VMPR compared to Luo and Angelidaki [16] (Table 3).

### 4.2. Implementation

Further development of the bioprocess technology is required before implementation. We observed low hydrogen conversion efficiencies during the optimization of VMPRs through increases in the gas influent rates. The efficiencies must, however, be improved so as not to lose valuable hydrogen gas into the effluent gas and ultimately into the natural gas infrastructure. Our study showed that improving these efficiencies by lowering the hydrogen influent rates resulted in considerably lower VMPRs. Therefore, the performance should be improved by other improvements. With the increased headspace pressure in Experiment 2, we already showed that increasing the hydrogen partial pressure improved these efficiencies. Experiment 2 also showed that with a pressurized atmospheric pressure (122 kPa), the VMPR increased at the highest hydrogen influent rate rather than decreased, which we observed for atmospheric pressures in Experiments 1 and 2 (Figure 3(a)). Pressurization, thus, reduced the hydrogen mass transfer limitations that became apparent because of the shorter hydrogen residence times in the bioreactors with an increased H_2_ influent rate. Further optimization studies with higher pressures are required though. Other measures to lengthen the gas residence times in the reactor vessel may also yield higher hydrogen conversion efficiencies by, for example, using a novel bioreactor configuration and by recycling effluent gas. The latter needs to be tested first as methane as an inert gas would decrease the hydrogen gas transfer flux as we have shown in Experiment 3. The resulting improved hydrogen conversion efficiencies must also result in the production of an effluent gas with a sufficient quality to introduce it into the natural gas grid. Here, we did not achieve such a quality, but with bioreactor design and operating condition improvements should be attainable while maintaining high VMPRs. In addition, before full-scale systems to upgrade biogas can be implemented, other research questions need to be answered, including what is the effect of a higher or lower (i) H_2_S concentration compared to the 7,000 ppm that was present in the industrial biogas here to test for possible sulfide toxicity or a deficiency of sulfur for metabolic growth, respectively and (ii) H_2_ : CO_2_ ratio compared to the 4 : 1 ratio that we used here?

To produce a high-methane-content renewable natural gas as our effluent gas, a source of sustainable hydrogen gas must be utilized. The first implementation of this technology will likely use off-peak electric power production to generate hydrogen gas via intermittent electrolysis of water. It is well known that methanogens can be intermittently fed and that dormant cultures can be started up rapidly in large-scale digester systems [2]. Current excess electric power exists in areas with a high density of wind or photovoltaic energy, and therefore, biogas upgrading can be incorporated into an energy storage system that transfers excess electric power from the existing electric grid into the other existing grid—the natural gas grid, which has a vast energy storage capacity. The overall electric-to-chemical energy conversion efficiencies are estimated to be ~60% when waste heat is not utilized and ~80% when waste is utilized. However, a detailed energy balance and life cycle assessment study is needed to ascertain the energy efficiencies and carbon dioxide recycling gains of storing energy rather than switching off the renewable generators such as windmills.

## 5. Conclusions

A thermophilic bioprocess with a pure culture of the hydrogenotrophic methanogen *M. thermautotrophicus* and with a continuous gaseous feeding scheme showed that the hydrogen gas transfer flux was limiting the conversion rates of carbon dioxide into methane. During an experimental period in which the hydrogen influent rate was increased, we observed increasing VMPRs of up to 49.2 L/L culture-day (1.33 g/L-h). However, this resulted also in lower hydrogen conversion efficiencies due to hydrogen bypassing. Therefore, careful optimization of the bioprocess is needed to maximize the hydrogen conversion efficiencies while maintaining a high enough conversion rate of carbon dioxide into methane. Pressurization of the headspace, indeed, increased the VMPR further to 65.6 L/L culture-day (1.79 g/L-h). Introducing methane into the influent gas with different relative ratios showed that methane itself was not inhibiting the conversion rates, but that it lowered the hydrogen partial pressures, resulting in lower methane production rates and hydrogen conversion efficiencies. The single culture of *M. thermautotrophicus* was also able to convert carbon dioxide from industrial, untreated biogas into methane with an external source of hydrogen gas. Chemical contaminants, such as hydrogen sulfide, and biological contaminants in biogas did not result in a reduction in performance (VMPR) even though some microbial contamination of the culture was observed. The biomass concentrations increased during the 8-day operating period to a maximum level of 8.0 g/L (OD_600_ of 15.2). The stable operating conditions, the robust activity, and the relatively high biomass concentrations under industrial conditions pave the way to develop this technology further to upgrade biogas into renewable natural gas when sustainable hydrogen is available.

## Figures and Tables

**Table 1:** The percentage of methane content in influent gas and total influent flow rate (*V*_in_) for Experiment 3. Three different methane influent rates (V_CH_4__) were evaluated for three different hydrogen influent rates (V_H_2__) while maintaining the stoichiometric 4 : 1 H_2_ : CO_2_ gas mixture ratio. The total influent flow rate (*V*_in_) is shown in brackets in L/min.

V_H_2__	V_CH_4__		
	0	0.2	0.4
0.2	0% (0.25)	44% (0.45)	62% (0.65)
0.4	0% (0.5)	29% (0.7)	44% (0.9)
1.6	0% (2)	9% (2.2)	17% (2.4)

**Table 2:** Average volumetric methane production rates and hydrogen conversion efficiencies for Experiment 1. Averages were taken for experimental values in Figure 2 at each hydrogen influent rate from 3 or 4 days. Standard deviations (SD) were calculated based on the values used to calculate the average.

H_2_ influent rate (L/min) (V_H_2__)	0.1	0.2	0.4	0.8	1.6
Number of data points	*n* = 3	*n* = 3	*n* = 4	*n* = 3	*n* = 3
Average VMPR (L/L culture-day)	9.93	18.5	34.0	47.9	40.4
SD	0.16	0.87	0.42	1.40	2.53
Average H_2_ conversion efficiency	96.6%	89.9%	82.7%	58.2%	24.5%
SD	1.60%	4.23%	1.02%	1.70%	1.54%

**Table 3:** Performance comparison between studies for *M. thermautotrophicus* cultures and reactor microbiomes that produce methane.

Studies*∖*parameters and references	Influent gas mixture	VMPR (L/L-day)	Fuel production rate (g/L-h)	Active volume (L)	References
Experiment 1	CO_2_/H_2_	49.2	1.33	3.5	This study
Experiment 2	CO_2_/H_2_	65.6	1.79	3	This study
Experiment 3	CO_2_/H_2_	50.5	1.37	3	This study
Experiment 3	Synth. biogas/H_2_	12.6	0.34	3	This study
Experiment 4	Industrial biogas/H_2_	12.0	0.32	3	This study
Pure culture *M. thermautotrophicus* study	CO_2_/H_2_	163	4.39	1.5	[21]
High-rate, laboratory-scale anaerobic digester	No infl. gas	11.7*	0.35	20	[24]
*In-situ* CO_2_ conversion with anaerobic digester	Industrial biogas/H_2_	0.45	0.01	3.5	[12]
Postprocessing with microbiome	Synth. biogas/H_2_	5.3	0.14	0.6	[16]

*VMPR was corrected for standard temperature and pressure.
